# Supplementary material for: Interactions between Epichloë endophyte and the plant microbiome impact nitrogen responses in host Achnatherum inebrians plants
Source: Microbiol Spectr. 2024 Mar 15;12(4):e02574-23. doi: 10.1128/spectrum.02574-23 (PMC10986526; doi:10.1128/spectrum.02574-23)
Supplement: Tables S1 to S3 and Figure S1 to S7 — Tables and Figures associated with microorganisms and metabolites of I and F plants. [file spectrum.02574-23-s0001.pdf]

Table S1 Total ion count and identification statistics table.

Ion mode	All peaks	Identified metabolites	Metabolites in Library	Metabolites in KEGG
pos	3003	580	519	159
neg	2991	198	157	71

Table S2 HMDB compound classification between endophyte-infected plants (I) and endophyte-free plants (F) under water (W) and ammonia-N (A) treatments.

Superclass	WN		UN		AN		NN	
	Number	%	Number	%	Number	%	Number	%
Lipids and lipid-like molecules	40	27.78	32	18.39	35	26.72	18	38.3
Organic acids and derivatives	38	26.39	75	43.1	38	29.01	13	27.66
Organoheterocyclic compounds	23	15.97	26	14.94	20	15.27	8	17.02
Phenylpropanoids and polyketides	12	8.33	6	3.45	9	6.87	3	6.38
Benzenoids	11	7.64	13	7.47	8	6.11	1	2.13
Organic oxygen compounds	10	6.94	14	8.05	14	10.69	2	4.26
Nucleosides, nucleotides, and analogues	5	3.47	3	1.72	2	1.53	2	4.26
Alkaloids and derivatives	3	2.08	3	1.72	5	3.82		
Hydrocarbon derivatives	1	0.69	1	0.57				
Organic nitrogen compounds	1	0.69	1	0.57				

Note: (1) Ion mode: the ion mode of the substance detected by the mass spectrometer, mainly: pos (positive ion mode) and neg (negative ion mode); (2) All peaks: the number of mass spectrometry peaks extracted by the software; (3) Identified metabolites: the number of metabolites finally identified by the primary and secondary mass spectrometry data (self-built libraries, Metlin, HMDB) (4) Metabolites in library: the number of metabolites annotated to public databases such as HMDB and Lipidmaps; (5) Metabolites in KEGG: the number of metabolites annotated to KEGG database.

Table S3 KEGG pathway between endophyte-infected plants (I) and endophyte-free plants (F) under water (W) and ammonia-N (A) treatments.

First Category	Second Category	WI_WF	AI_AF
Metabolism	Amino acid metabolism	10	14
	Biosynthesis of other secondary metabolites	3	6
	Carbohydrate metabolism	1	0
	Lipid metabolism	2	6
	Metabolism of cofactors and vitamins	3	3
	Metabolism of other amino acids	1	2
	Metabolism of terpenoids and polyketides	2	2
	Nucleotide metabolism	4	3
Environmental Information Processing	Membrane transport	6	1
	Signal transduction	0	2
Genetic Information Processing	Translation	0	2

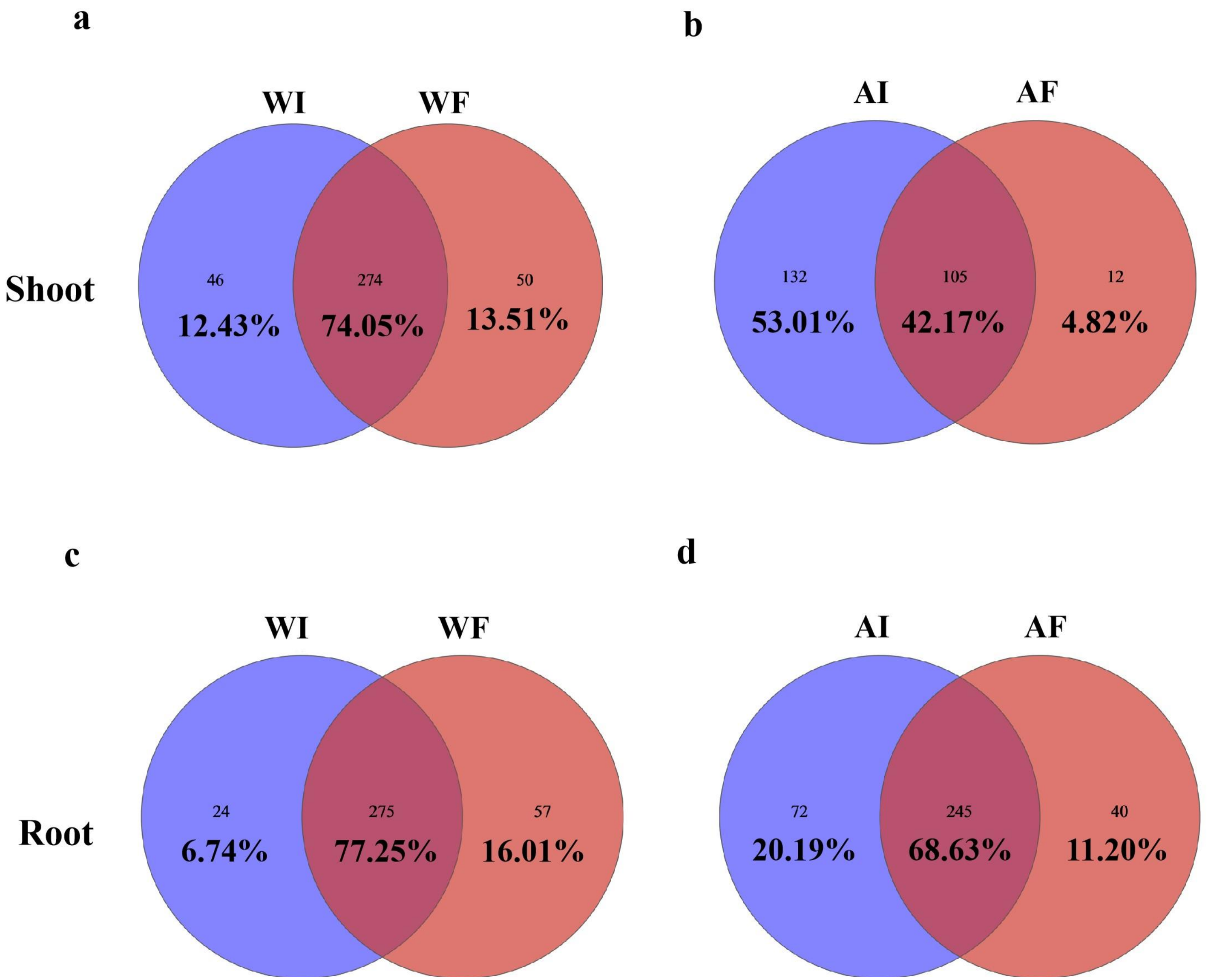

Fig.S1 Venn plot showing number of common and unique OTUs in shoot (a and b) and root (c and d) of bacterial genera between endophyte-infected plants (I) and endophyte-free plants (F) under water (W, a and c) and ammonia-N (A, b and d) treatments.

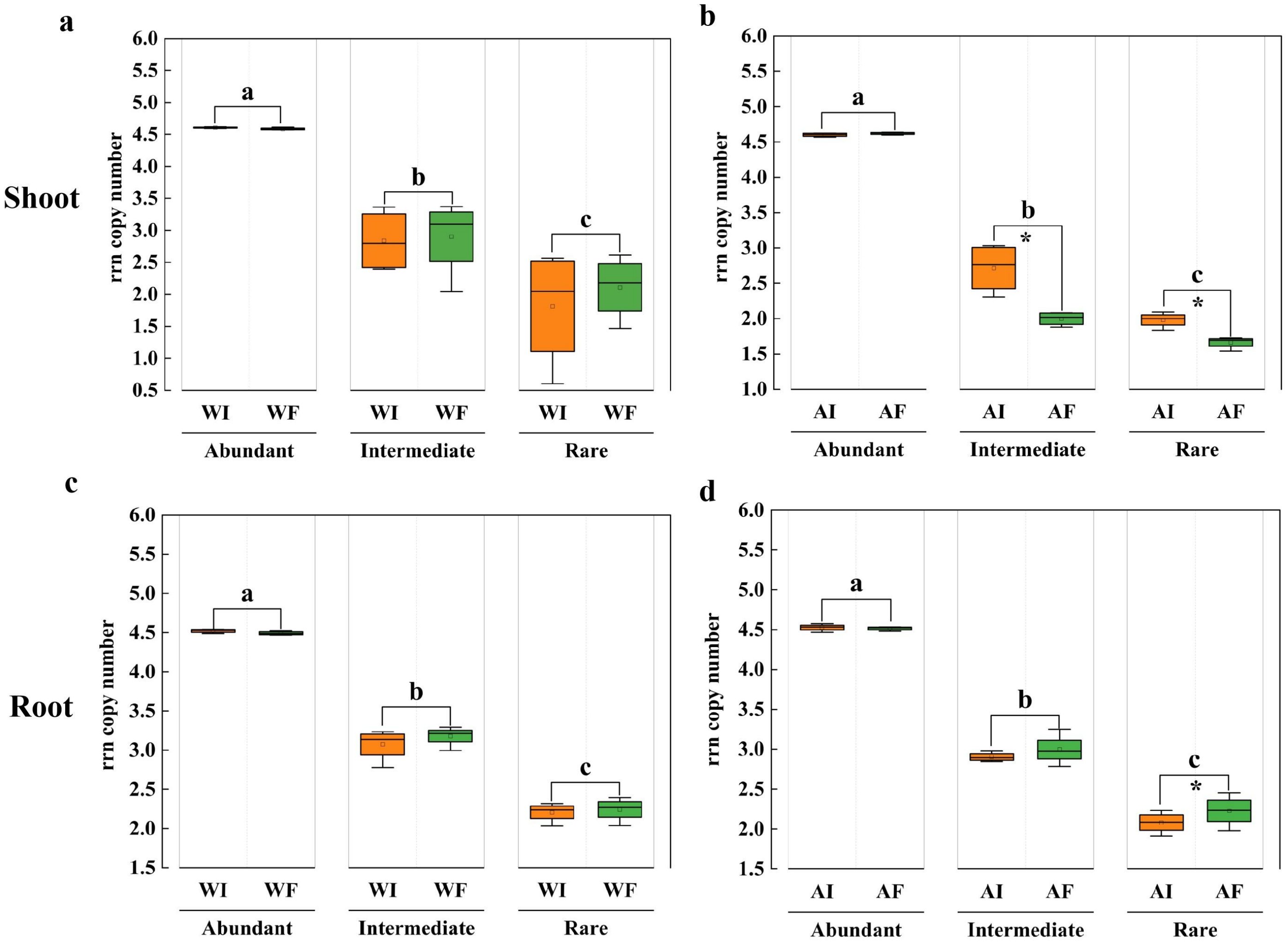

Fig.S2 (a to d) Differential abundance analysis (DAA) of OUT relative abundance at abundant, intermediate and rare bacterial community in shoot (a and b) and root (c and d) between endophyte-infected plants (I) and endophyte-free plants (F) under water (W, a and c) and ammonia-N (A, b and d) treatments.

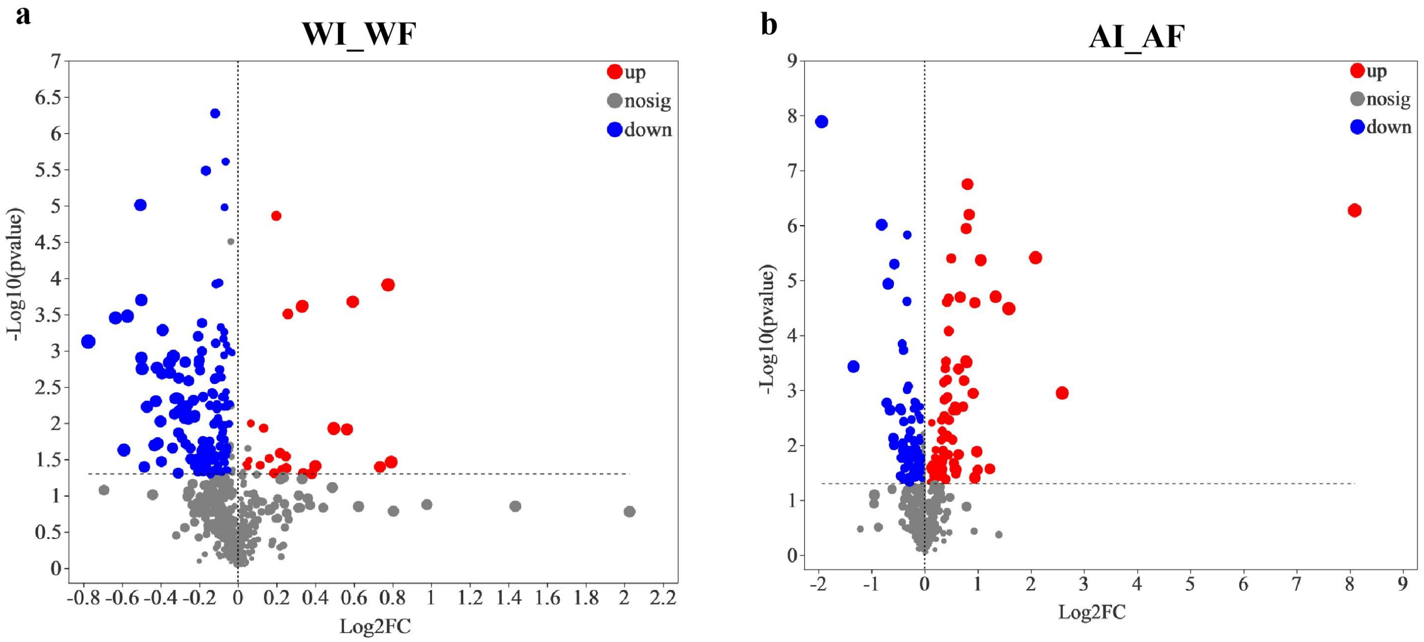

Fig.S3 Volcano plot of metabolite expression differences between endophyte-infected plants (I) and endophyte-free plants (F) under water (W, a) and ammonia-N (A, b) treatments. The horizontal coordinate is the value of the fold change in the difference in

expression of the metabolite between the two groups, i.e. log2FC. The vertical coordinate is the statistical test value for the difference in metabolite expression change, i.e. -log10(p_value) value, with higher values indicating more significant expression differences. Each point in the graph represents a specific metabolite and the size of the point indicates the Vip value. The points on the left are metabolites with down-regulated expression differences and the points on the right are metabolites with up-regulated expression differences, with the more significant expression differences the further to the left, right and top the points are.

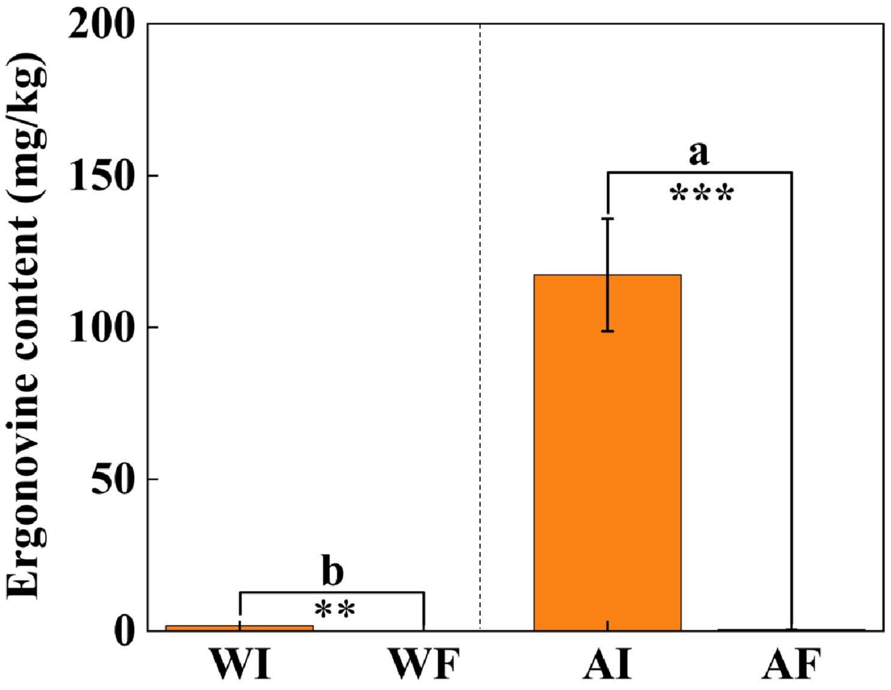

Fig.S4 Ergonovine content in shoot of endophyte-infected plants (I) and endophyte-free plants (F) under water (W) and ammonia-N (A) treatments.

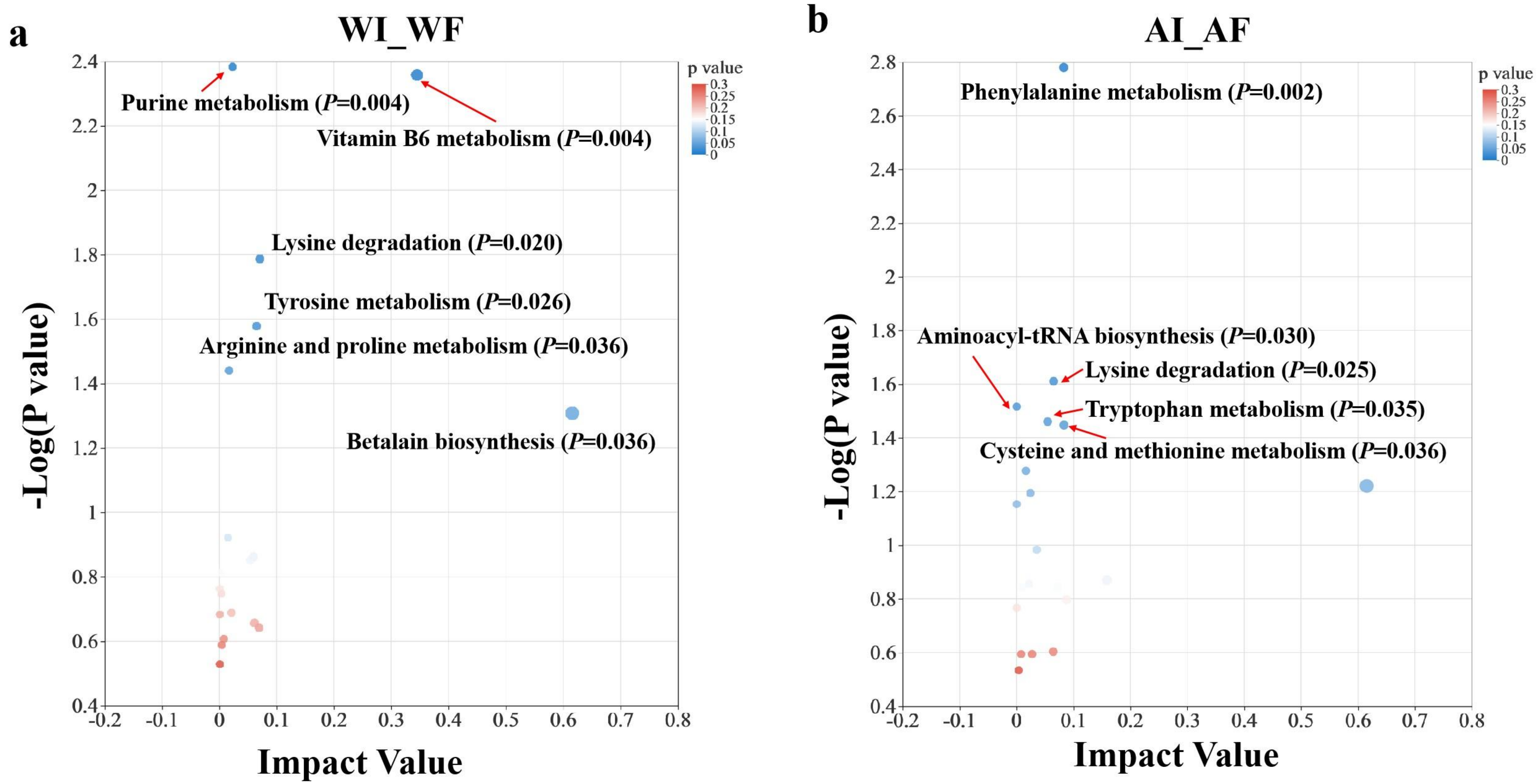

Fig.S5 (a and b) KEGG topology analysis by relative-betweenness centrality methods showing pathways with significant metabolite enrichment in root exudates of endophyte-infected plants (I) and endophyte-free plants (F) under water (W, a) and ammonia-N (A, b) treatments. Each bubble: a KEGG Pathway. X-axis: the relative importance of metabolites in the pathway, impact value. Y-axis: the enrichment significance of the metabolite involvement pathway, -log10(*P* value). Bubble size: impact value. The bubble with larger, the importance of pathway with greater.

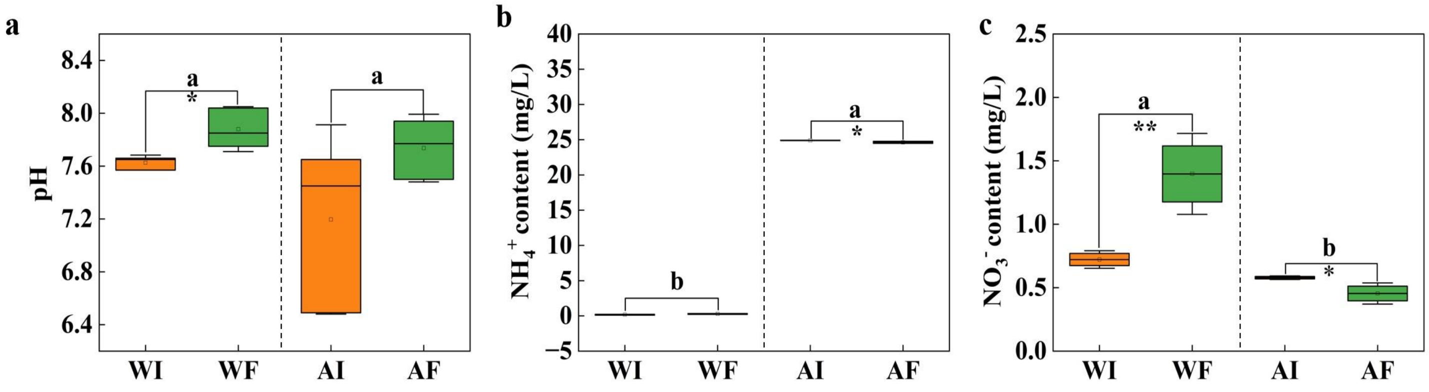

Fig.S6 KEGG enrichment analysis showing a pathway of significant metabolite enrichment between endophyte-infected plants (I) and endophyte-free plants (F) under water (W) and ammonia-N (A) treatments. X-axis: the enrichment significance *P*-value. Y-axis: the KEGG pathway. The size of the bubbles represents how much of the pathway is enriched into the metabolic compound; the smaller the p-value, the more statistically significant it is, generally *P*-value less than 0.05 is considered as a significant enrichment term for this function

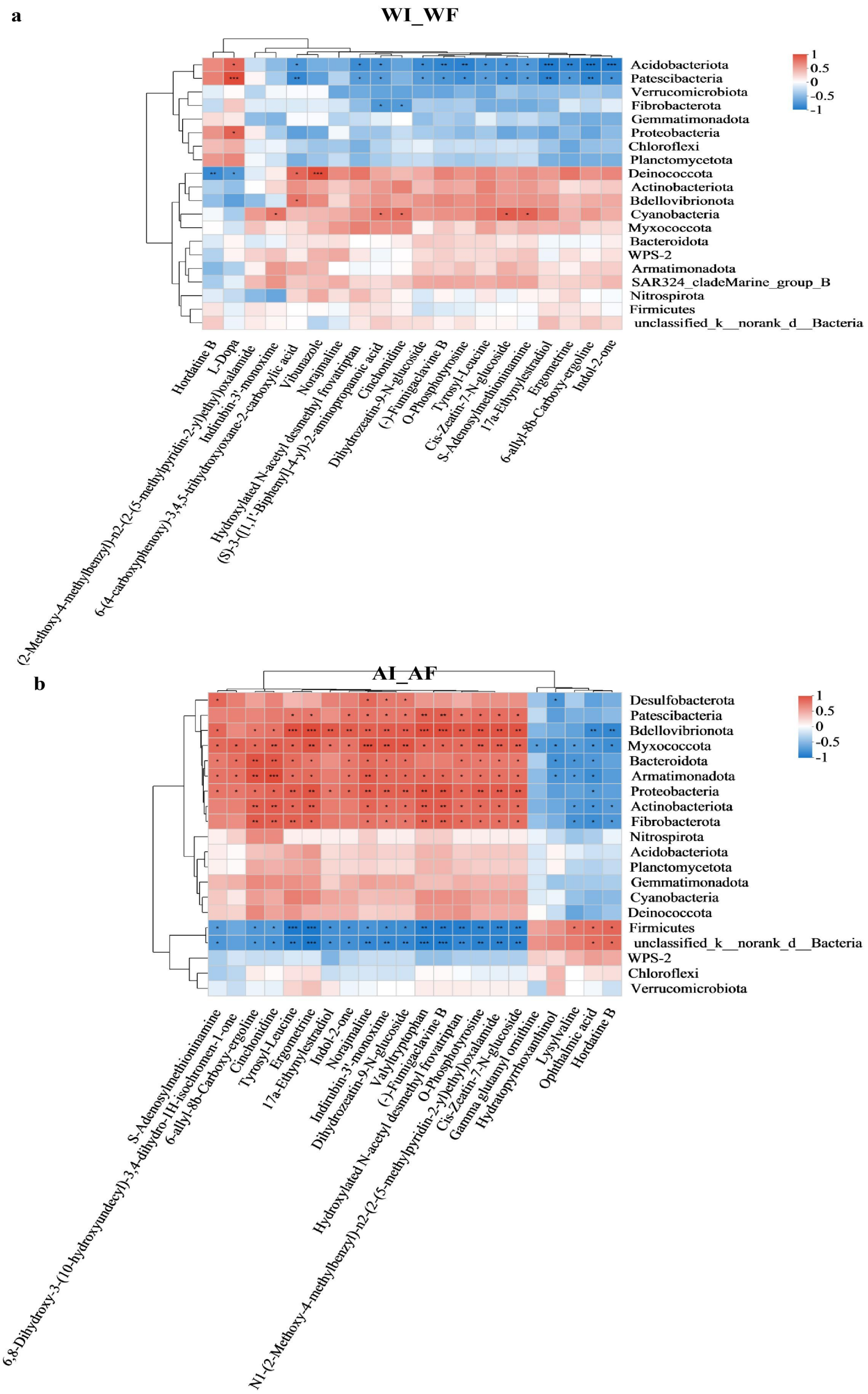

Fig.S7 Correlation heatmap plots visualizing the relationship between root bacterial microbial community and metabolic compounds of root exudates in endophyte-infected plants (I) and endophyte-free plants (F) under water (W, a) and ammonia-N (A, b) treatments. X-axis: metabolites. Y-axis: bacterial phyla community. Different colors: correlation R-value. If the *P*-value is less than 0.05, it is marked with *; right side legend, color intervals for different R values; *, $P \leq 0.05$; **, $P \leq 0.01$; ***, $P \leq 0.001$.
